# Supplementary material for: Optimization of acquisition time and reconstruction parameters for quantitative single-photon emission computed tomography/computed tomography using iodine-123 metaiodobenzylguanidine
Source: PLoS One. 2025 Sep 30;20(9):e0330655. doi: 10.1371/journal.pone.0330655 (PMC12483245; doi:10.1371/journal.pone.0330655)
Supplement: Attachment — Submitted filename: Response_to_Reviewers_R1.docx [file pone.0330655.s003.docx]

**Our point-by-point response to all comments and suggestions is listed below:**

**Response to Reviewers**

PONE-D-24-40275

Optimization of acquisition time and reconstruction parameters for quantitative single-photon emission computed tomography/computed tomography using iodine-123 metaiodobenzylguanidine

PLOS ONE.

Dear Editor,

We appreciate your review of our manuscript. In response to your comments, we have carefully revised our manuscript within the limitations of a *Report Article*. Our responses are provided below; the revisions are indicated in red in the revised manuscript. Deleted parts are indicated as strikethrough and in blue.

We have made minor adjustments to the graph's formatting to eliminate unnecessary white space.

Thank you again for your comments on our manuscript. We hope that the revised manuscript is now acceptable for publication as a *Report Article*.

Sincerely.

**Response to Reviewer #1**

PONE-D-24-40275

Optimization of acquisition time and reconstruction parameters for quantitative single-photon emission computed tomography/computed tomography using iodine-123 metaiodobenzylguanidine

PLOS ONE.

We appreciate your review of our manuscript. In response to your comments, we have carefully revised our manuscript within the limitations of a *Report Article*. Our responses are provided below; the revisions are indicated in red font in the revised manuscript. Deleted parts are indicated as strikethrough and in blue.

We have made minor adjustments to the graph's formatting to eliminate unnecessary white space.

Thank you again for your comments on our manuscript. We hope that that the revised manuscript is now acceptable for publication as a *Report Article*.

Line 27: *“single-photon emission computed tomography/computed tomography images were acquired by varying the acquisition time per detector” what does “per detector” mean, isn’t it rather the total acquisition time of the image volume?*

Response: Thank you for your query. As you correctly pointed out, the total acquisition time for the image volume is determined by the product of "time per view per detector" and "number of views," divided by the "number of detectors." Given this, we have revised the terminology into "time per view" to avoid any potential reader misunderstandings. (Line 28)

Lines 40-42: *“In conclusion, an acquisition time of ≥50 s/view with an SI product of approximately 120 and a Gaussian filter of 10−12 mm is desirable for evaluating the myocardium and surrounding tissues as background” It is the H/M ratio that allows us to judge the estimation of myocardial activity relative to the surrounding, this ratio was not considered in this study. This conclusion should be better specified.*

Response: Thank you for your valuable comments. Our study aimed to optimize imaging conditions for accurate SUV analysis of 123I-MIBG SPECT using phantoms with clinically relevant radioactivity concentrations. Although we did not directly compare our findings with the H/M ratio in this study, our results could provide a foundation for future clinical applications where more accurate myocardial uptake assessment is required.

As you correctly pointed out, the H/M ratio is a critical parameter for evaluating the relative activity between the myocardium and surrounding tissues. However, the conventional H/M ratio is susceptible to influences from surrounding organs such as the lungs and liver. Additionally, the H/M ratio can be significantly affected by the choice of region of interest. Three-dimensional evaluation can overcome these limitations by providing a more accurate assessment of anatomical overlap.

Although we did not directly compare the myocardial and surrounding tissue uptake in clinical cases using MIBG, studies using FDG-PET have demonstrated the utility of SUV ratios between the myocardium and surrounding tissues, as discussed in our response to Lines 68-70.

In future studies, we plan to build upon these findings by conducting clinical studies to investigate the relationship between the optimized imaging parameters and H/M ratio. Specifically, we aim to clarify the relationship between the H/M ratio and disease progression or type in patients with dementia, with the goal of improving diagnostic accuracy and clinical utility.

Owing to the abstract's word limit, we indicated that comparing our results to the H/M ratio is a goal for future clinical applications.

“*In conclusion, an acquisition time of ≥50 s/view with an SI product of approximately 120 and a Gaussian filter of 10−12 mm is required for measuring SUVs with ^123^I-MIBG. The results of this study provided a foundation for future research comparing our findings with the H/M ratio, thus advancing the clinical application of our method.*” (Lines 42-44)

Line 40. *“For ≥50 s/view, the variations in maximum and mean SUVs decreased. The relative standard error was <5%. » No explicit error analysis supports this claim, especially regarding mean SUVs.*

Response: Thank you for your comment. We have revised Fig 6 to make it easier to highlight what the mean SUVs are in the Results section instead of the Abstract. I will provide a detailed answer to the question on Line 192 about the revision of Fig 6.

Lines 68-70: *« Since the basic verification of the comprehensive relationship between the myocardium and surrounding tissues has not been clarified, further research is required for clinical application », the manuscript raises an important issue that does not seem directly related to the study, as it does not provide any clarification on this issue*

Thank you for your valuable comments. Although the clinical utility of MIBG SPECT in patients with dementia remains to be elucidated, studies using FDG-PET have demonstrated that assessing the SUV ratio between the myocardium and surrounding tissues can improve the detection accuracy of hypermetabolic regions in cardiac sarcoidosis compared with evaluating the myocardium alone [12,13]. Our study aimed to optimize parameters for quantitative SPECT analysis of 123I-MIBG. In addition, we suggest that future studies considering the uptake in both the myocardium and surrounding tissues could have the potential for establishing more accurate diagnoses.

We have added references to other studies specifically addressing quantitative SUV analysis to support our claims.

“*In cardiac sarcoidosis studies using fluorodeoxyglucose-positron emission tomography, the relationship between the myocardium and surrounding tissues has been investigated. In addition, it has been reported that using the SUV ratio with the aorta as a reference allows for more accurate assessment of high-uptake areas compared with evaluating the myocardium alone [12,13]. However, using ^123^I-MIBG SPECT, the basic verification of the comprehensive relationship between the myocardium and surrounding tissues has not been clarified. Further research on routine clinical application of ^123^I-MIBG SPECT is required.*” (Lines 74-82)

[12] Manabe O, Ohira H, Yoshinaga K, Naya M, Oyana N, Tamaki N. Qualitative and quantitative assessments of cardiac sarcoidosis using 18F-FDG PET. Ann Nucl Cardiol. 2017; 3:1117–1209.

[13] Tsujimoto M, Fukushima A, Kawai H, Watanabe M, Tanahashi S, Sarai M, et al. Volume-based 18 F-FDG PET analysis of cardiac sarcoidosis using the descending aorta as a reference tissue. Nucl Med Commun. 2023;44:390-396.

Lines 71-73: *» Therefore, in this study, we aimed to build evidence for clinical application and conduct a basic study on the convergence and quantification of images for the acquisition and reconstruction conditions for measuring SUV. »*

*How does this study provide clinical evidence? This is not the aim of the study, no clinical evaluation was conducted.*

Response: Thank you for your comment. Although our study does not involve clinical cases, we suggest that our findings could contribute to establishing parameters for accurately assessing hypometabolic regions in conditions such as cognitive disorders. To address this, we have revised the statement as follows:

"*Therefore, this study aimed to establish evidence for accurately quantifying SUV in ^123^I-MIBG SPECT/CT studies. This is particularly in cases of decreased myocardial uptake, such as in cognitive disorders, by conducting a basic study on the convergence and quantification of images for the acquisition and reconstruction conditions for SUV measurement.*"(Lines 83-88)

Line 133, *equation 1 requires further explanation.*

Response: Thank you for your comment. We have added a clarification to make the indicator in Equation 1 more intuitive.

“*Using an ideal system, all RC_j_ would equal 1. However, due to partial volume effects, actual systems exhibit smaller RC_j_ values. Integrating 1-RCj over the range between 7 and 30 mm in Equation (1), we could quantify the system's resolution. A higher RC_t_ indicated a lower resolution.*” (Lines 159-162)

Lines 191-192: *"The SUVmax and SUVmean results for each hot sphere when the Gaussian filter was changed are shown in Fig 6" This is not correct, Fig. 6 only shows the SUVmax.*

Response: Thank you for your comment. We initially decided to show only the SUVmax values in the figures to avoid visual clutter. However, we appreciate your insightful feedback.

We have now added the mean SUV values to Fig 6. Furthermore, regarding the mean SUV, we have included the standard deviation of the measurement values obtained at the reference acquisition time of 100 s/view as a relative standard error. (Fig 6)

Lines 193-195: "the relative error rates of the SUV at each time point were 25.86% and 13.99% for the SUVmax and SUVmean at 10 s/view, 13.20% and 195 8.92% at 30 s/view, and 5.47% and 3.94% at 50 s/view, respectively." These error rates cannot be deduced from Fig. 6. More precision is needed on the calculation of these error rates.

Response: Thank you for your valuable comments. We have revised Fig 6 based on your insightful suggestions. We have modified the figure to ensure that each mean value plot is clearly distinguishable. (Fig 6)

**Response to Reviewer #2**

PONE-D-24-40275

Optimization of acquisition time and reconstruction parameters for quantitative single-photon emission computed tomography/computed tomography using iodine-123 metaiodobenzylguanidine

PLOS ONE.

We appreciate your review of our manuscript. In response to your comments, we have carefully revised our manuscript within the limitations of a *Report Article*. Our responses are provided below; the revisions are indicated in red in the revised manuscript. Deleted parts are indicated as strikethrough and in blue.

We have made minor adjustments to the graph's formatting to eliminate unnecessary white space.

Thank you again for your comments on our manuscript. We hope that the revised manuscript is now acceptable for publication as a *Report Article*.

-Line 59: *The authors state that “does not eliminate the influence of overlap”. They should better precise what they mean here. Overlap between which structures?*

Response: Thank you for your comment. Due to the anatomical overlap of the heart with surrounding organs such as the liver and lungs, accurately quantifying myocardial activity can be challenging. We have revised the text to clarify this point.

“*The H/M ratio is easy to measure. However it is limited by the influence of overlap of surrounding tissues such as the liver and lungs, making accurately assessing local myocardial function difficult*.” (Lines 61-64)

-Line 63: *The same holds true for this sentence. They should precise what they mean by “distribution of accumulation.”.*

Response: 3D SPECT imaging offers a more accurate assessment of radionuclide distribution in organs and tissues by eliminating the overlap issues associated with 2D planar imaging. We have revised the text to improve readability.

“*three-dimensional evaluation using SPECT allows for a more accurate assessment of radionuclide distribution in organs and tissues by eliminating the overlap issues associated with planar imaging.*” (Lines 65-68)

-Lines 82-83: *more information about the NEMA IEC Body phantom should be provided, e.g. company, site, country and/or internet site*.

Response: Thank you for your feedback. We have added information on the purchase source of the Phantom.

“*the NEMA IEC Body phantom (AcroBio Corp., Tokyo, Japan)*” (Line 102)

-Introduction: *The authors should specify why they chose these phantoms. Do they realistically represent organ structures? Why not using e.g. a four-dimensional NURBS-based cardiac-torso (NCAT) phantom, which provides a realistic model of the normal human anatomy and cardiac and respiratory motions?*

Response: Thank you for your valuable comment.

The 3D-OSEM is known to be sensitive to variations in pixel values depending on the counts contributing to the image and reconstruction parameters (Jpn J Radiol Technol 2012; 68:686–695). Rod phantoms, a traditional evaluation method, have been used for assessing in-plane image characteristics by eliminating partial volume effects between slices, a fundamental characteristic of SPECT systems. However, the rod-shaped design is more industrial in nature. Moreover, clinical practice often involves spherical or irregular shapes where partial volume effects between slices must be considered. For this reason, NEMA IEC body phantoms are widely accepted in PET.

In this study, rather than evaluating the impact of respiratory motion or blurring due to cardiac pulsation as suggested in the comments, we focused on determining the extent to which quantitative accuracy could be achieved with a stationary structure that simulated the actual uptake of myocardial and other tissues. We have clarified the optimization process as a series of steps.

Although our previous research has optimized parameters for Tc-99m, discussions about low radioactivity levels, such as background noise, those for I-123 have not been addressed. We anticipate that future studies will focus on harmonization with other vendors and the characteristics of MIBG for staging.

The selection of the phantom is described in the "Materials and Methods" section under "Equipment and Phantom."

“*Due to the lower resolution of SPECT images compared with positron emission tomography images and fluctuation of three-dimensional ordered subset expectation maximization (3D-OSEM) reconstruction of factors such as counts and reconstruction parameters, we evaluated image quality using phantoms with rod and spherical structures, as previously reported [14, 15].*” (Lines 96-100)

-Lines 89-94: *Please specify the reason for the choice of the settings of the main energy window, the sub-energy windows, the matrix size, the zoom, the sampling angle and the acquisition time per detector. Does the choice correspond to typical settings of clinical applications? How do the settings vary between examinations of the heart and the brain?*

Response: This study focused on the thoracic region, including the heart. In contrast to brain SPECT, where the target is the cerebral cortex with a thickness of 3-4 mm and pixel size is ideally set to 2 mm or less based on the sampling theorem, this study does not target the brain region. Therefore, we have set the necessary pixel size and sampling angle based on the myocardial thickness and used a standard protocol optimized for the iodine-123 energy peak. We have added references regarding SPECT acquisition and CT conditions.

“*The SPECT parameters were set according to a standard protocol based on myocardial thickness, including pixel size, sampling angle, and energy peak-based optimization of the iodine-123 [16]*.” (Lines 108-110)

[16]Dorbala S, Di Carli MF, Delbeke D, Abbara S, DePuey EG, Dilsizian V, et al. SNMMI/ASNC/SCCT guideline for cardiac SPECT/CT and PET/CT 1.0. J Nucl Med. 2013;54:1485-1507.

-Lines 101-108: *The same question holds for the parameters of the CT acquisitions – are they routinely used and how they vary for the different body areas to examine?*

Response: In addition to the reference [16], we added that the CT images were taken while the parameters were set according to the vendor's recommended conditions.

“The CT parameters were set according to the vendor's recommendations.” (Line 122)

-Discussion section: *The authors should briefly discuss how patient body mass may affect the image quality and quantification accuracy of 123I-MIBG SPECT images. Would their conclusions hold for higher body mass?*

Response: We appreciate your valuable feedback. This study optimized acquisition time and reconstruction parameters using a phantom designed to simulate a standard-sized individual (60 kg) injected with a full dose of 111 MBq. As you correctly pointed out, directly applying the results of this study to individuals with significantly different body sizes or to regions where the administered radioactivity dose differs for the same examination may introduce bias into the study. However, we suggest that the results of this study can still be useful for determining acquisition time and reconstruction parameters under different conditions by calculating the reference radioactivity concentration per tissue. To enhance the clarity of results, we have added the following limitation to the discussion section:

“*This study optimized acquisition time and image reconstruction parameters based on a phantom simulating a standard-sized individual (60 kg) injected with a full dose of 111 MBq of ^123^I-MIBG. Therefore, applying these results to settings with different administered radioactivity doses or patient body sizes, considering the radioactivity concentration per tissue is necessary.*” (See. Limitations)

*-Although supporting data have been provided, they cannot be read. Probably they can only be read by the appropriate Siemens software.*

Response: The supporting data can be read using imageJ, RAVAT or Siemens software.

To facilitate data access, the folder structure has been streamlined and is detailed in the accompanying Readme file. Download links to the necessary software are included.

Minor comments:

-Line 57: *should be “… (ROI) in the myocardium …*”

Response: Thank you for your comments. We have revised the manuscript according to your suggestions. (Line 59)

-Line 70: s*uggest rewriting to “... is required for routine clinical application of 123I-MIBG SPECT.”.*

Response: Thank you for your comments. We have revised the manuscript according to your suggestions. (Lines 81-82)

-Line 125: *Suggest rewriting the caption of figure 1 to “JS-10 phantom”.*

Response: We have made the exact revisions you kindly suggested. (Line 150)

-Line 293: *should be “Acknowledgements”*.

Response: We have made the exact revisions you suggested.

-*The unit in figure 2 is 15.81 kBq/ml.*

-*Throughout the manuscript, please change the unit of milliliters to ml (SI unit).*

Response: Thank you for your comment.

The International System of Units (SI) permits the use of both "l" and "L" as symbols for the liter. However, to avoid confusion with the numeral "1", the 16th General Conference on Weights and Measures (CGPM) recommended using "L" (Metrologia, 1980, 16, 56-57). Therefore, we have uniformly used "L" throughout the revised version of the manuscript.

Nevertheless, we are open to adhering to specific guidelines or requirements by individual countries or journals.
