# Supplementary material for: Optimization of acquisition time and reconstruction parameters for quantitative single-photon emission computed tomography/computed tomography using iodine-123 metaiodobenzylguanidine
Source: PLoS One. 2025 Sep 30;20(9):e0330655. doi: 10.1371/journal.pone.0330655 (PMC12483245; doi:10.1371/journal.pone.0330655)
Supplement: Attachment — Submitted filename: 1750161253294-Response3Engv3.docx [file pone.0330655.s004.docx]

**Our point-by-point response to all comments and suggestions is listed below:**

**Response to Editor**

PONE-D-24-40275R1

Optimization of acquisition time and reconstruction parameters for quantitative single-photon emission computed tomography/computed tomography using iodine-123 metaiodobenzylguanidine

PLOS ONE

Dear Editor,

We appreciate your review of our manuscript. In response to your comments, we have carefully revised our manuscript following the guidelines for the Report Article. Our responses are provided below; the revisions are indicated in red in the revised manuscript, while the deleted parts are indicated as strikethrough and in blue.

Thank you again for your comments on our manuscript. We hope that the revised manuscript is now acceptable for publication as a Report Article.

Sincerely.

**Response to Reviewer 3**

We appreciate your review of our manuscript. In response to your comments, we have carefully revised our manuscript following the guidelines for a Report Article. Our responses are provided below; the revisions are indicated in red font in the revised manuscript.

Deleted parts are indicated as strikethrough and in blue.

Thank you again for your comments on our manuscript. We hope that that the revised manuscript is now acceptable for publication as a Report Article.

**Major comments:**

First, only the hot rod region was evaluated in the NEMA and JS-10 phantoms (Disc 2). It is important to evaluate the defect in the myocardial MIBG phantom. Hence the need for a cold rod evaluation would be necessary. In particular, the JS-10 phantom can be verified similar to the MIBG defect evaluation by incorporating the radioactivity concentration of the myocardium in the BG and the radioactivity concentration of the mediastinum in the Rod. This evaluation should be added.

**Response:**

Thank you for this important comment on the evaluation of defects, particularly intra-myocardial cold spots. We acknowledge the clinical significance of assessing such defects in MIBG scintigraphy.

However, the primary objective of our study was to optimize acquisition and reconstruction parameters for the accurate quantification of uptake values (SUV), focusing specifically on ensuring the stability and quantitative accuracy of both the low-uptake background region (simulating the mediastinum) and high-uptake target regions (simulating myocardial tissue). Therefore, the evaluation of localized intra-myocardial defects, as suggested, was outside the primary scope of this investigation. To clarify the trajectory of our research aims, we have revised a sentence in the **Introduction** section.

*Further research, including foundational studies to define optimal acquisition and reconstruction parameters for SUV analysis, is essential to support the routine clinical use of ^123^I-MIBG SPECT.* (Page5, Lines 95-98 of the revised manuscript with track changes)

Regarding your kind suggestion to configure the JS-10 phantom with the background (BG) at a myocardial uptake level and the rods at a lower (e.g., mediastinal) uptake level for defect evaluation: We suggest that the large size of the JS-10 phantom, which does not closely mimic the dimensions of the human heart within the torso, would lead to imaging counts that are not representative of clinical conditions if the entire BG were filled to myocardial activity levels. This could create a mismatch with the count statistics relevant to optimizing parameters such as the SI product for clinical cardiac SPECT.

Our evaluation using Disc 3 of the JS-10 phantom (a water insert in a uniform radioactive background) was intended to assess the system's ability to accurately represent regions with known zero radiopharmaceutical uptake and characterize BG noise and scatter (via SR and CV). We agree that this is different from evaluating intra-myocardial "*cold defects*." However, we suggest that an accurate scatter ratio (SR) assessment, which reflects the correct delineation of such regions with zero uptake, indirectly contributes to the proper evaluation of true "*cold spots*" when using iterative reconstruction methods by ensuring better background characterization and contrast. To avoid confusion with intra-myocardial defects specifically, we have strived to use the term "*cold spot*" more consistently in our Discussion when referring to regions of low or absent uptake in a broader sense. We have revised the **Discussion** and the **Summary** **and Implications** sections to more precisely reflect this intent and the revised terminology.

*RC_t_ serves as an index for evaluating high-uptake regions, while SR reflects background regions with no radiopharmaceutical accumulation. An accurate SR indirectly supports reliable evaluation of true defects, as cold region visibility is particularly important when using iterative reconstruction methods [14].* (Page 17, Lines 356-368)

*Our study findings suggest that, for accurate evaluation of linearity in high uptake regions, reliable representation of cold regions in the background...*(Pages 21-22, Lines 474-464)

Second, this study does not simulate the human body. In particular, MIBG has a high accumulation in the liver. The study does not consider the effect of scattered rays from the liver. You mention the limitations of the scatter correction in your discussion as well. Therefore, it is uncertainty whether this phantom result can be clinically useful. The fact that the scattered rays from the liver affect especially in the mediastinum must also be considered in your novelty. At least a clinical evaluation should be added to show the validity of this condition.

**Response:**

Thank you for your insightful comment on the importance of scatter from the liver. We agree that this is a key limitation of our phantom-based approach and that our standardized phantoms cannot fully replicate the complex inter-organ scatter found in clinical settings.

In response to your feedback, we have substantially revised the "**Limitations**" section to explicitly address this point. The revised text now clearly states that our phantom setup does not account for scatter and septal penetration effects from high-uptake organs like the liver. Furthermore, we have added a caution that the clinical application of our findings, particularly for mediastinal SUV, warrants further validation in more realistic settings such as anthropomorphic phantoms or clinical studies.

We are confident that this revision transparently defines the scope and applicability of our work for the reader, thereby fully addressing your important concern.

Third, our phantom setup does not account for the complex scatter and septal penetration effects originating from high-uptake organs adjacent to the heart, most notably the liver. This is a critical factor in clinical settings that can influence the quantification of nearby low-uptake regions such as the mediastinum. For these reasons, caution is warranted when generalizing our findings. The clinical application of these optimized parameters, particularly for mediastinal SUV, should be validated in settings involving different patient body sizes, administered doses, or through studies using more anthropomorphic phantoms. (Pages 21, Lines 452-460)

Finally, your English text is poor and contains many errors, including multiple definitions of abbreviations. If you wish to resubmit your paper, we strongly recommend that you proofread it in English.

**Response:**

Thank you for your direct comment on the English language quality of our manuscript and the issues with abbreviations. We sincerely apologize for any errors or lack of clarity in the previous version.

To address this significant concern, we have taken the following steps:

Professional English Proofreading: The entire revised manuscript, including the abstract, main text, figures, tables, and references, has been thoroughly proofread and edited by a professional English language editing service specializing in scientific manuscripts (Editage, a service employing native English-speaking editors with expertise in life sciences/medical research). We believe that this has significantly improved the grammar, clarity, style, and overall readability of the paper.

Review and Correction of Abbreviations: We have meticulously reviewed all abbreviations used throughout the manuscript. We have ensured that:

Each abbreviation is defined at its first use in both the Abstract and the main text, as appropriate.

Abbreviations are used consistently after their initial definition.

Any instances of multiple or inconsistent definitions for the same abbreviation have been corrected.

We have also checked for any undefined abbreviations.

We are confident that these comprehensive revisions have addressed the issues you raised concerning the English text and use of abbreviations. We hope that the improved clarity and accuracy of the language now make the manuscript suitable for further consideration.

Other points of concern:

Line 58

Add the full spelling of H/M

**Response:**

Thank you for pointing this out. We have added the full spelling for the H/M ratio at its first appearance in both the Abstract and the main text.

In the **Abstract** (Page 3, Lines 51-52), it now reads: '*...*c*omparing this method with theheart-to-mediastinum ratio...*'

In the main text (Page 4, Lines 69-70), it now reads: ' *Conventionally, the semiquantitative index known as the heart-to-mediastinum (H/M ratio)—..*. **'**

Line 103-105

You have determined the SPECT parameters based on Reference 16. However, its guidelines are standard protocols by MPI. SPECT parameters by Tc and Tl differ from those by I-123 in terms of dose, accumulation rate, and collection counts. A standard protocol for MIBG has already been proposed in the EANM. If your protocol differs from that standard protocol, you should either re-experiment or consider the impact of the differences in discussion.

In particular, matrix and pixel size are significantly different.

**Response:**

Thank you for your pertinent comment on the justification of our SPECT parameters and their relationship with established guidelines, particularly those from thee EANM. We have carefully considered your points and revised the manuscript to provide a clearer rationale for our chosen parameters.

Our SPECT parameter selection was informed by general principles of cardiac SPECT imaging, as outlined in the SNMMI/ASNC/SCCT guideline [16], and by specific recommendations for^123^I-MIBG SPECT. In the revised Materials and Methods section, we have now additionally cited the EANM 2012 guidelines for radionuclide imaging of phaeochromocytoma and paraganglioma (Taieb D, et al.), which recommends a 128 x 128 matrix for ^123^I-MIBG SPECT acquisition, aligning with our choice.

We acknowledge that the EANM procedural guidelines for cardiac sympathetic imaging with ^123^I-MIBG (Agostini D, et al. 2010) suggests a 64 x 64 matrix for routine clinical use. To address this difference and justify our selection of a 128 x 128 matrix (3.90 mm pixel size), we have added a specific discussion point to the Discussion section. This new paragraph elaborates on our rationale, emphasizing the aim of enhancing quantitative accuracy by potentially mitigating partial volume effects, while also acknowledging the need for considering these parameter differences when extrapolating findings to different clinical protocols.

The revised text in the **Materials and Methods** section (Page 7, Line 151) now includes the citation of the Taieb D, et al. guideline.

The new discussion paragraph can be found in the **Discussion** section (Pages 20-21, Lines 429-438) and reads as follows:

*The 128 × 128 matrix (3.90 mm pixel size) employed in this study offers higher spatial resolution than the 64 × 64 matrix recommended by the EANM guidelines for cardiac sympathetic imaging [26]. This choice was deliberately made to enhance the accuracy of quantitative SUV assessment—a primary objective of this research—by mitigating partial volume effects and enabling a more precise delineation of radiopharmaceutical distribution. Our findings suggest that even with smaller pixel sizes, appropriate optimization of acquisition time and 3D-OSEM reconstruction parameters can yield stable quantitative values. However, it is important to consider these parameter differences when applying these findings to routine clinical practice, which may follow different standard protocols.*

Line 112-113

You set the SI product to 60, 120, and 180. The evidence for this set up should be provided. The 3D-OSEM with Flash3D algorithm has a proposed SI product of 90-120 in MPI. However, MIBG imaging is not sure if it is the same protocol due to low acquisition counts. In particular, the SI product of OSEM affects spatial resolution and image uniformity, and is also involved in the accuracy of SUVs. The relevance of the SI product should be mentioned in your paper.

**Response:**

Thank you for your valuable feedback regarding our choice of SI products. We agree that the rationale for selecting these values should be justified within the manuscript.

In response to your comment, we have expanded the **Discussion** section to more thoroughly explain the significance of the SI product range we investigated. The revised text now clarifies that our finding of SI 120 as optimal is consistent with prior studies. Furthermore, we explain that investigating the broader range, including SI 60 and 180, was crucial for demonstrating the negative impacts of under-convergence and noise amplification, respectively, which contextualizes our choice of the optimal value.

We believe this addition to the **Discussion** provides the clear justification you requested and strengthens the rationale of our study.

T*his optimal SI product of 120 is optimal aligns with these previous reports. The broader range investigated in our study was crucial for demonstrating this optimum: an SI product of 60 yielded poorer quantitative accuracy due to under-convergence, while an SI product of 180 increased image noise without a corresponding benefit to SUV recovery, a particular concern for low-count ^123^I-MIBG imaging*. (Page 20, Lines 420-425)

Line 117-123

You indicated two different CT reconstruction parameters. What does this mean? Do you evaluate the effect of attenuation correction due to differences in CT imaging conditions? You should add details on how you utilized these two parameters. If there is only one type of reconstruction protocol used as attenuation correction, one or the other should be removed as they are not relevant to the paper.

**Response:**

Thank you for your comment on the CT reconstruction parameters. We apologize for the previous lack of clarity.

We have revised **the Materials and Methods section** to specify only the CT acquisition and reconstruction parameters used for SPECT attenuation correction, as this was the sole application of CT relevant to our SPECT optimization study. The description about a second '*diagnostic*' CT reconstruction has been removed to improve conciseness and relevance.

The revised text now clearly states the single set of CT parameters used for attenuation correction, including the use of the B08s smoothing function.

*'CT images for attenuation correction were acquired according to the vendor's recommendatio, using a tube voltage of 130 kV, ref. mAs of 50 mAs (care dose type: AEC mean), a rotation time of 1.5 s, beam collimation of 6 x 2.0 mm, and pitch of 1. The images were reconstructed using the high-frequency smoothing function B08s with a slice thickness of 4 mm, a reconstruction increment of 4.0 mm, and a field of view of 650 mm (voxel size: 1.27 × 1.27 mm)*. (Page 8, Lines 166-175)

Line 135-136

You set the radioactivity concentration ratio of Rod to BG to 10. The reasons for this setup are important. You also mention in your objectives the accuracy of quantitation in low-accumulation cases. Please provide additional examination of the effect of different radioactivity concentration ratios on count recovery. Or discuss the influence of count recovery in your discussion with reference to previous studies.

Response:

Thank you for your valuable comment regarding the rationale for our chosen radioactivity concentration ratio and its influence on count recovery. We agree this is an important point and appreciate the opportunity to clarify our experimental design.

We would like to clarify that our study did, in fact, assess the impact of varying concentration ratios on quantitative accuracy. In our dose linearity (DL) evaluation using **Disc 1**, the rod-to-background (Rod:BG) ratio was systematically varied from 2:1 up to 16:1. As presented in our results, this evaluation confirmed the robustness of our quantitative methods across this wide range of clinically relevant contrast levels.

Furthermore, the specific physical relationship between varying ratios and the Recovery Coefficient (RC) is a topic we have investigated in detail in our previous work [19], which is cited in the manuscript.

Building on these findings, the RC evaluation in the current study (using Disc 2) was designed with a specific focus. We deliberately used a fixed 10:1 ratio to test our optimized 123I-MIBG SPECT protocol under a challenging but clinically plausible scenario of moderately reduced myocardial uptake. This approach allowed us to perform a detailed assessment of partial volume effects under this specific condition, rather than repeating an extensive analysis of varying ratios which we had already characterized.

To make this rationale and the context of our study clear in the manuscript, we have revised **the Discussion section**. The revision now explicitly explains the distinct roles of our Disc 1 (varying ratios) and Disc 2 (fixed ratio) evaluations and clarifies how this work builds upon our prior research [19].

We believe this clarification fully addresses your concern by demonstrating that the influence of different concentration ratios was indeed considered and appropriately handled within our study design.

*For this RCt evaluation, we used a fixed 10:1 ratio to simulate a moderately reduced myocardial uptake, building upon our prior work that characterized the physics of RC dependency across various ratios [19].* (Page 17, Lines 353-356)

*Second, the optimization was based on a phantom simulating a standard-sized individual (60 kg) who received a full dose of 111 MBq of ^123^I-MIBG. The activity concentrations used reflect clinically plausible scenarios: the background (mediastinal) concentration (1.42**–1.73 kBq/mL) accounted for approximately 20–30% early excretion of the administered MIBG and assumed uniform distribution of the remaining activity [18,20]. The hot sphere concentration (15.81 kBq/mL) was based on an estimated 1–2% of the effective in-body MIBG activity accumulating in the myocardium [27,28].* (Page 17, Lines 445-452)

Line 139

The full spelling of ROI has already been described in Line 59.

**Response:** Thank you for noticing this redundancy. We have corrected Line 139 (Page 9, Line 194 of the revised manuscript with track changes).

Line 145-146

You have evaluated the correlation between the SPECT counts and the actual radioactivity concentration. Is the actual radioactivity concentration image-based or dose calibrated? Since this study is based on quantitation, the DL should be calculated between Bq/mL of dose calibrator and Bq/mL of image-based Bq/mL

**Response:**
Thank you for this critical question regarding our dose linearity (DL) assessment. We agree that the ultimate goal of quantitation is to establish a linear relationship between true and image-derived Bq/mL values.

Our study employed a deliberate, stepwise methodology. As you correctly observed, the initial evaluations using the JS-10 phantom, including the DL assessment, were performed on a SPECT-count basis. This was an intentional choice.

The primary purpose of this initial phase was to screen and optimize the reconstruction parameters before proceeding to absolute SUV quantification. To this end, we followed the established methodology of previous foundational studies [14, 15], which utilize a series of count-based physical indices (DL, RC_t_, SR, CV) to assess the intrinsic performance of the reconstruction algorithm. This approach allows for a robust evaluation of linearity and spatial resolution, free from the potential confounding variables of a BCF, which can itself be imprecise under not-yet-optimized, noisy conditions.

To ensure this rationale and our overall study design are transparent to the reader, we have added a new introductory paragraph at the beginning of the "**Evaluation using the JS-10 phantom**" subsection within the **Discussion**. The new introductory paragraph now explicitly states that the JS-10 phantom evaluations served as this crucial, count-based parameter optimization phase.

We are confident that this single, comprehensive revision clarifies the context for all our JS-10 analyses. It explains why the DL was assessed on a count basis, why a relative RC was used (your comment on Lines 147-149), and how Figure 3 should be interpreted, thereby fully addressing this series of important concerns.

*The evaluations using the JS-10 phantom served as a crucial initial phase to screen and optimize reconstruction parameters. Following the methodology of previous foundational studies [14, 15], we utilized a series of SPECT-count-based physical indices—specifically DL, RC_t_, SR, and CV—before proceeding to SUV quantification with the NEMA phantom. This stepwise approach allowed us to assess the intrinsic performance of the reconstruction algorithm under various conditions. For DL assessment, we evaluated the system's fundamental proportional response. The DL was excellent under all conditions, with correlation coefficients ranging from 0.996 to 0.999.* *This indicates that, regardless of acquisition time or reconstruction parameter, or the rod-to-background ratio (which ranged from 2:1 to 16:1), the 3D-OSEM algorithm accurately represented the relative radioactivity concentrations between high-uptake and background regions. These findings align with previous studies using technetium-99m [14,19], suggesting that a practical convergence point had been achieved for accurately reflecting the proportional relationship between high-uptake areas and background activity.* (Page 16-17, Lines 333-349**)**

Line 147-149

You are calculating the relative recovery factor, but the 30mm diameter rods are already affected by the partial volume effect. It should either be an absolute recovery factor or a relative recovery factor with the outer container without Disc containing the same radioactivity concentration as Hotrod as a reference. The relative recovery coefficient does not reveal the percentage of each Rod that underestimates the quantitative value.

**Response:**

Thank you for your insightful comment regarding the methodology of our recovery coefficient (RC) evaluation.

As you correctly point out, the 30 mm rod is subject to partial volume effects, and our RC is therefore a relative, not an absolute, measure. This was an intentional choice within our stepwise study design.

As we have now clarified in a new introductory paragraph i**n the Discussion's "Evaluation using the JS-10 phantom**" subsection, all evaluations with this phantom were designed as a crucial initial phase to screen reconstruction parameters using SPECT-count-based physical indices. This established methodology [14, 15] allows for the robust comparison of parameter settings without the confounding variables of absolute calibration, which is performed in the subsequent NEMA phantom stage.

Therefore, the use of a relative RC, with the 30 mm rod as an internal reference, fits perfectly within this framework. Its purpose is not to measure absolute recovery, but to assess how different parameters affect the relative spatial resolution, which is reflected in the shape of the recovery curve.

We believe that the new introductory paragraph in our **Discussion**, which explains the overall count-based rationale for the JS-10 evaluations, comprehensively addresses this concern. By clarifying that all indices in this phase (including RC) are part of a relative, comparative analysis, the context and purpose of our chosen RC methodology are made clear to the reader. (Page 16-17, Lines 333-349**)**

Line 163-164

How do you consider this relationship of radioactivity concentration in relation to your research objectives? Some reports on MIBG SUVs have already been published by Sito S et al as follows. Your determination of radioactivity levels should be set up with clinical feedback. In particular, the radioactivity concentration is important in relationship to the spill-in and spill-out effects.

Saito S, et al. Absolute quantitation of sympathetic nerve activity using [123I] metaiodobenzylguanidine SPECT-CT in neurology. EJNMMI Rep. 2024; 8(1): 15.

Saito S, et al. Three-Dimensional Heart Segmentation and Absolute Quantitation of Cardiac 123I-metaiodobenzylguanidine Sympathetic Imaging Using SPECT/CT. Ann Nucl Cardiol. 2023; 9(1): 61-67.

**Response:**

Thank you for your comment and for suggesting these additional references. We have carefully reviewed the two papers by Saito et al. and appreciate you bringing them to our attention.

We agree that these are valuable contributions to the field. However, our study's primary focus is on the foundational technical optimization of SPECT parameters, whereas the suggested papers concentrate on the downstream clinical application and interpretation of MIBG SUV.

As per the journal's guidance on citation, we have evaluated the relevance of these works to our specific study. While they are important for the broader field, we are concerned that a detailed discussion of these clinical studies would detract from the core technical focus of our manuscript. Our goal is to maintain a clear and focused narrative on the physics and parameter optimization, which we believe serves as a necessary prerequisite for the type of clinical applications described in the suggested papers.

The rationale for the radioactivity concentrations used in our phantom has been clarified in the "**Limitations**" section, based on our aim to simulate plausible biodistribution scenarios.

Therefore, to preserve the scientific clarity and focus of our work, we have respectfully chosen not to incorporate these citations. We hope you understand our reasoning.

Line 177 Table 1

Correlation coefficients are calculated in Table 1, but there is no description of the statistical method. Which is Pearson correlation coefficient or Spearman('s) rank-correlation coefficient? It is necessary to show whether there is a significant relationship for all correlations.

**Response:**

Thank you for your comment on the statistical method used for the correlation coefficients in **Table 1** and the need to report their significance.

The correlation coefficients (DL (r)) presented in Table 1 for the dose linearity assessment with the JS-10 phantom are Pearson's correlation coefficients. To address your comment and provide clarity on our statistical approach, we have made the following revisions to the manuscript:

First, we have added a new subsection titled "**Statistical Analysis**" to **the Materials and Methods section**. This new subsection describes the statistical methods used, including the use of Pearson's correlation coefficient for the DL evaluation and the software used for analysis. The added text for this subsection is as follows:

***Statistical Analysis***

*Continuous variables, including SPECT count data and calculated SUVs, were analyzed using descriptive statistics and are presented as mean ± standard deviation (SD).*

*For DL evaluation using the JS-10 phantom, Pearson's correlation coefficient was calculated to assess the relationship between the actual radioactivity concentrations in the rods and the average SPECT counts (R1–R4). A p-value of < 0.01 was considered statistically significant. All statistical analyses were conducted using BellCurve for Excel (Social Survey Research Information Co., Ltd., Tokyo, Japan).'* (Page 11, Lines 239-245)

Second, we have updated the description accompanying **Table 1** in **the Results section**.

***'****Pearson's correlation coefficient (r) was >0.99 with a p-value <0.01 for all DL assessments, indicating a strong and statistically significant positive correlation.****'***(Page 12, Lines 255-25)

Line 196

the larger the RCt were ?

**Response:**
Thank you for pointing out the ambiguity in our original phrasing regarding the relationship between the Gaussian filter, SI product, RCj, and RCt. We intended to describe how changes in the Gaussian filter and SI product affect RCj, which in turn influences RCt. We have revised the sentence to clarify this relationship.

The revised sentence in **the Results section** (Pages 12-13, Lines 270-274 of the revised manuscript with track changes) now reads:

' *Comparing the results (Fig 4 and Table 1) for a given acquisition time, a larger full width at half maximum of the Gaussian filter or a smaller SI product generally resulted in lower RC_j_ for individual rods, and consequently, higher RC_t_*.

Line 203-204

I don't understand if it's an “decrease was gradual” or not due to four measurement points only.

**Response:**

Thank you for this valid point regarding our use of the term 'gradual' to describe the decrease in CV with only four measurement points. We agree that a more objective and data-driven description is appropriate.

Upon further detailed analysis of the CV data presented in **Table 1**, particularly examining the rate of change between the four acquisition time points, we have a clearer understanding of the trend. We have revised the sentence to more accurately reflect our observations.

*The CV decreased with longer acquisition times.* *Notably, a substantial reduction in CV was observed when the acquisition time increased from 10 s/view to 30 s/view, whereas further increases to 50 s/view and 100 s/view resulted in comparatively smaller decreases. In contrast, the SR decreased as the acquisition time increased up to 100 s/view.* (Page14, Lines 281-285)

This revised phrasing accurately conveys that the most significant improvement in CV occurred within the shorter acquisition time range (10 to 30 s/view), and the benefit of further extending the acquisition time beyond that, in terms of CV reduction, was less pronounced. We hope that this adequately addresses your comment on the characterization based on a limited number of data points.

Line 208-209

While it is important to consider spatial resolution and CV, it is also important to consider whether the point of convergence of the 3D-OSEM method calculation has been achieved.　However, you have not shown the convergence point of the 3D-OSEM method. Please indicate if this is also a reasonable point of convergence for successive iterative reconstruction.

**Response:**

Thank you for your question regarding the convergence of the 3D-OSEM method in the context of our NEMA phantom parameter selection.

You are correct that ensuring a sufficient degree of convergence is critical for reliable quantification. The parameters chosen for the NEMA phantom evaluation were determined based on the comprehensive, multi-metric assessment performed with the JS-10 phantom, as previously discussed (your comment on Line 145-146).

Our approach was to identify the optimal balance among these competing indices (DL, RCt, SR, and CV). The excellent dose linearity, for instance, suggests the reconstruction had achieved a state stable enough to maintain a proportional response. We consider this overall stability across multiple metrics to be an indicator of practical convergence for quantitative purposes.

To make the basis of our parameter selection clearer, we have revised the relevant sentence in the manuscript. The new text now explicitly states that the NEMA study parameters were chosen based on the optimal balance of these quantitative indices from the JS-10 evaluation.

We believe this clarification, which highlights our reliance on a comprehensive assessment to ensure practical convergence, fully addresses your concern. A more detailed discussion of this concept is provided later in t**he Discussion section** (your comment on Line 236-239).

Line 213-216

You show the SUVs in each sphere for each collection time. However, you have not described the results.

Response:

Thank you for pointing out the omission in describing the SUV results shown in Figure 6. We have added a detailed description about these findings to **the Results section**.

The revised **Results section** now includes the following description about the SUVmax and SUVmean values observed in Figure 6:

*The SUVmax and SUVmean of each hot sphere under different Gaussian filter settings are presented in Fig 6. Using the 100 s/view acquisition as the reference standard, SUVmax for each sphere fluctuated as acquisition time decreased. This fluctuation lessened with the application of a wider Gaussian filter, though both SUVmax and SUVmean tended to decrease. Relative error rates, calculated using the SUV values at 100 s/view as the reference standard, were 25.86% and 13.99% for SUVmax and SUVmean, respectively, at 10 s/view; 13.20% and 8.92% at 30 s/view; and 5.47% and 3.94% at 50 s/view.* (Page 15, Lines 302-309)

We hope that this addition adequately described the results shown in Fig 6.

Figure 3

The linearity between the dose calibrator and the Bq/mL on the SPECT image is important, as mentioned above.

**Response:**

Thank you for this follow-up comment. We agree this is an important point for clarifying the interpretation of Figure 3.

As detailed in our response to your earlier comment (regarding Lines 145-146), Figure 3 intentionally plots SPECT counts on the Y-axis. Its specific purpose is to demonstrate the system's fundamental proportional response, which is the prerequisite for applying a BCF for absolute quantification.

To ensure this context is immediately clear to any reader viewing the figure, we have revised the caption for Figure 3. **The new caption** now explicitly states that the Y-axis represents SPECT counts and clarifies that the plot's purpose is to validate this proportional response.

We believe this direct clarification in the figure caption, combined with the broader context provided in our revised Discussion, fully addresses your concern.

***Fig. 3. Results of dose linearity.*** *This count-based assessment was performed to validate the proportional response of the system, a prerequisite for SUV quantification.* （Lines 264-265）

Line 220-222

There is no explanation for a, b, and c in the figure. In addition, what does the 100s/view error bar mean? Is it a relative error? We think the error bars in this figure are inappropriate. If it is common practice to show error bars for relative errors in the figure, please provide a rationale for this.

Thank you for your valuable feedback on Figure 6. You correctly pointed out the confusing discrepancy between the labels (a, b, c) in the figure and the "Left/Center/Right" description in the caption. We also agree that the error bars were inappropriate and potentially misleading. We apologize for this lack of clarity.

To address your comments and improve the overall readability of the figure, we have made the following key revisions:

Figure Layout and Labels: We have revised the layout of Figure 6 to make the relationships between the different filter settings clearer. In line with this, we have removed the unnecessary (a, b, c) labels, ensuring the figure now directly corresponds to the caption's description.

Error Bars: The error bars originally shown represented the intra-VOI standard deviation of pixel values, not inter-measurement variability. We agree with you that this was not an appropriate representation of error in this context. Therefore, to ensure scientific rigor, we have removed all error bars from the revised figure.

The revised Figure 6, along with its updated caption, now presents the SUVmax and SUVmean trends in a much clearer and more consistent manner. We are confident that these substantial improvements fully address your concerns.

***Fig 6.*** ***SUVmax and SUVmean for each hot sphere when the Gaussian filter is changed****.*

*(Left column) Gaussian filter: 8 mm; (Center column): 10 mm;(Right column): 12 mm.*

*(Top row) SUVmax; (Bottom row) SUVmean.*

*The red dashed line shows the theoretical SUV. SUV, standardized uptake value*

*(Lines 311-316)*

Line 226

Please include references.

**Response:**

Thank you for pointing this out. We have now added appropriate citations **[20, 21]** to support the statement, as requested.

*21. Nakajo M, Horizoe Y, Kawaji K, Jinguji M, Tani A, Fukukura Y, et al. Application of 123I-MIBG myocardial maximum standardized uptake value to characterize cardiac function in patients with pheochromocytoma: comparison with echocardiography. Jpn J Radiol. 2023;41: 437-448.*

Line 230-232

Background level is important, but the relationship between cardiac accumulation and background is also important in quantitative evaluation. You have failed to evaluate that point in detail.

**Response:**

Thank you for emphasizing the importance of the relationship between cardiac and background uptake. We fully agree that this relationship is central to quantitative evaluation.

This point was also raised in your previous comments (regarding Lines 135-136 and 163-164). In response, we have substantially revised the manuscript to clarify the rationale for our phantom's design.

Specifically, the "**Limitations**" section now provides a detailed explanation of how our radioactivity concentrations were derived from the literature to simulate clinically plausible target-to-background ratios. Furthermore, our evaluation of dose linearity across a wide range of ratios (2:1 to 16:1) with the JS-10 phantom, and our subsequent SUV and CNR analysis with the NEMA phantom, were all fundamentally designed to assess quantitative performance under these critical target-to-background conditions.

Therefore, we are confident that our study not only acknowledges the importance of this relationship in its design but also evaluates it in detail. The revisions made to the manuscript now make this transparent, fully addressing your concern.

Line 236-239

DL is only linearity, and it is inappropriate to evaluate the convergence of image reconstruction parameters.

**Response:**

Thank you for your insightful comment, which raises an important point about the terminology of "convergence" in the context of dose linearity (DL). We appreciate the opportunity to clarify our perspective and its basis in the literature.

We agree that DL does not directly measure the mathematical convergence of pixel values with increasing iterations in the strictest sense. However, in our study, we use DL as a powerful indicator of what we refer to as **"practical quantitative convergence."** This concept, consistent with the approach in prior foundational studies [14], defines convergence not by pixel-wise stability, but by the point at which the reconstruction provides a quantitatively stable and reliable output for clinical purposes.

Our rationale is that if an iterative reconstruction process has not sufficiently converged, the resulting image will contain unstable noise and artifacts. This instability would inherently disrupt the strict proportional relationship between true activity concentrations and measured signals, leading to poor dose linearity.

Therefore, the fact that we consistently observed excellent linearity (correlation coefficients > 0.99) across a wide range of acquisition and reconstruction parameters is strong evidence that the 3D-OSEM algorithm had reached a state stable enough for robust quantitative analysis.

This line of reasoning is already reflected in our manuscript. As stated in our **Discussion section**, these findings "*align with previous studies using technetium-99m [14,19], suggesting that a* ***practical convergence point had been achieved*** *for accurately reflecting the proportional relationship between high-uptake areas and background activity*."(Page 17, Lines 343-346).

We believe this clarification of our definition of "practical convergence," and its justification based on both our results and the cited literature, fully addresses your concern.

Line 249-250

To corroborate the abrupt noise, you should add the resulting SUV variation in the sphere.

**Response:**

Thank you for your suggestion to provide data corroborating our discussion on "abrupt noise." We agree that this point requires clear, data-driven evidence.

For this purpose, we believe that the CV, which we have already presented in Table 1, serves as the most direct and appropriate metric to quantify statistical noise in our JS-10 phantom evaluation. While SUV variation within a sphere can also reflect noise, it is a more complex index influenced by other factors such as partial volume effects and signal recovery. In contrast, the CV measured in the uniform background region provides a purer assessment of the statistical noise itself.

Our data in Table 1 clearly shows that the CV increases significantly as acquisition time decreases, providing direct evidence for the "abrupt noise" at shorter scan times.

To make this link explicit and directly address your concern, we have revised the relevant sentence in our Discussion. The updated text now clearly states that the observed reversal in RCt at short acquisition times is attributable to the higher statistical noise, as evidenced by the increased CV shown in Table 1.

We are confident that this approach—linking the phenomenon directly to our established and appropriate noise metric (CV)—provides the robust evidence you requested and fully clarifies the basis for our interpretation. The revised discussion now reads:

***'***T*he convergence of RCt towards the true system resolution critically depends on applying a sufficiently high SI product, which in turn requires an adequate number of acquired counts [22,23]. The observed reversal in RC_t_ at shorter acquisition times is likely due to the reduced number of photons contributing to image formation, resulting in a higher CV (i.e., greater statistical noise), which could adversely affect RC_t_ measurements. However, it is important to note that simply increasing the SI product to improve RC_t_ convergence can also lead to increased CV if not supported by sufficient count statistics.****'*** (Page 18, Line 368-375)

Line 284-285

You emphasize that the evaluation of background equivalent to the mediastinum is an advantage over other studies. However, its clinical significance has not been described. Hence, the importance of optimizing image reconstruction processing conditions at the BG level has not been demonstrated. You should discuss the clinical significance of this study.
Response:

Thank you for this important comment regarding the clinical significance of optimizing parameters at the background (BG) level. We agree that clarifying this link is crucial.

Our study is a foundational technical investigation, not a clinical validation study, so we cannot directly demonstrate a new clinical utility. However, the clinical significance of our work lies in enhancing the reliability of existing and emerging quantitative metrics, such as the well-established H/M ratio and SUV measurements.

The accuracy and reproducibility of any such metric that uses the mediastinum as a reference are fundamentally dependent on the stable and accurate quantification of the BG region itself. If the BG signal is noisy or biased due to suboptimal imaging parameters, the resulting H/M ratio or myocardial-to-mediastinal SUV ratio will be inherently unreliable.

Therefore, our work—by identifying the optimal parameters for accurately quantifying these low-uptake BG regions—provides the essential technical foundation required to improve the precision and stability of these clinically significant indices.

To ensure this point is explicitly clear to the reader, we have added a statement to our "**Summary and Implications**" section. This new text clarifies that a key clinical implication of our findings is the potential to enhance the robustness of established comparative metrics like the H/M ratio, thereby supporting more reliable quantitative cardiac assessments in the future.

We believe this addition directly addresses your concern by explicitly stating the clinical relevance of our technical findings.

***'****The clinical implication of these findings is significant for the advancement of quantitative MIBG SPECT. By establishing technical parameters that ensure the stable and accurate quantification of not only high-uptake myocardial regions but also low-uptake background areas like the mediastinum, our work provides an essential foundation for enhancing the robustness of 3D-based quantitative metrics, such as the myocardial-to-mediastinal SUV ratio. This, in turn, has the potential to support more reliable and reproducible cardiac assessments in clinical practice.* ***'***(Page 23, Lines 480-487)

Line 290-292

The evidence of radioactivity concentration is insufficient. You have determined the radioactivity concentration from previous study. How much of this do you assume accumulated in the myocardium and blood pool in patients who received MIBG? It is not possible to determine whether the radioactivity concentration settings in this study are related to the accumulation of myocardium and blood pools by the clinical cases. Your study especially emphasizes the importance of optimizing Bq/mL in the mediastinum. The radioactivity concentration in the mediastinal part of the MIBG should be measured in several cases and the mean and SD should be added.

**Response:**

Thank you for this important follow-up comment regarding the evidence for our chosen radioactivity concentrations.

We fully agree that linking phantom settings to clinical reality is a critical aspect of this research. As this topic was also raised in your previous comments (on Lines 135-136 and 163-164), we have substantially revised the manuscript to clarify the rationale for our chosen concentrations. Specifically, the revised **Limitations section** now details how the activity levels were derived from established literature [18, 20, 27, 28] to simulate plausible clinical scenarios, including our assumptions about administered dose, patient body size, early excretion, and typical myocardial and mediastinal uptake.

Regarding your suggestion to measure radioactivity in several clinical cases, we recognize this as a valuable undertaking for a future clinical validation study. However, the primary objective of our current investigation was to establish optimal technical parameters using a standardized, reproducible phantom setup. A prospective clinical study to establish a reference range for mediastinal radioactivity would be a logical next step but falls outside the scope of this foundational technical work.

Therefore, we are confident that the revisions made—which transparently explain the basis of our phantom settings—appropriately address the core of your concern within the defined scope of our study.

Line 303

We have not been able to evaluate your results regarding the visualization of defects. Also, Disc 3 is inappropriate as a defect evaluation.

If a defect evaluation is to be performed, a cold rod evaluation should be performed in Disc 1 and Disc 2.

**Response:**

Thank you for your comment on our results on the "*visualization of defects*" and the appropriateness of Disc 3 for such an evaluation.

As addressed in detail in our response to your first major comment (**Major Fault 1**), the term "*visualization of defects*" has been replaced with "*the accurate representation of non-radioactive regions within the background*" (see revised **Summary and Implications**).

This revised wording accurately reflects that our evaluation with Disc 3 (the water insert) was focused on assessing the system's ability to delineate known zero-activity areas within a radioactive background and characterize background uniformity and noise (via SR and CV). We agree that Disc 3 is not a model for intra-myocardial cold defects.

Therefore, we hope that with this clarification of terminology, your concern regarding our results on "visualization of defects" and the role of Disc 3 has been addressed. The manuscript now precisely describes what was evaluated in this context.

Line 314

The acquisition time is also important, but since the results are only for this phantom, it would be easier for the reader to understand if the myocardial and mediastinal counts were shown as Projection counts.

**Response:**

Thank you for your valuable suggestion to provide projection count data, which would indeed help readers relate our phantom-based findings to count levels typically encountered in clinical practice.

We agree that presenting projection counts could offer a more tangible measure of the statistical level of our acquisitions. To address this, we have now included representative projection data files (JS-10_ECT_tomo.IMA and nema_ECT_tomo.IMA) for both the JS-10 phantom and the NEMA IEC body phantom, respectively, as **Supplementary Material**. These projection data are provided for context and have been added to the end of the **Results section**

This supplementary data will allow readers to directly compare the count statistics obtained in our phantom experiments with those from their own clinical ^123^I-MIBG SPECT acquisitions, thereby facilitating a better understanding about the applicability of our optimized parameters.

**Response to Reviewer 4**

We appreciate your review of our manuscript. In response to your comments, we have carefully revised our manuscript following the guidelines for a Report Article. Our responses are provided below; the revisions are indicated in red font in the revised manuscript.

Deleted parts are indicated as strikethrough and in blue.

Thank you again for your comments on our manuscript. We hope that that the revised manuscript is now acceptable for publication as a Report Article.

1- Line 62 – Blood pool can also overlap with the myocardium.

**Response:**

Thank you for this important point. We agree that the blood pool is a significant surrounding tissue that could overlap with the myocardium. We have revised Line 62 to include the blood pool as one of the overlapping tissues.

Revised manuscript: Page4, Line 72-75 of the revised manuscript with track changes:

*H/M ratio is easy to measure;* *however, it is limited by overlapping signals from surrounding tissues, such as the liver, lungs, and blood pool, which complicate accurate assessment of regional myocardial function*.

2- Line 91 – Lower values indicate better resolution, so the term “poorer” should be used instead of “lower.” Also, specify that this refers to spatial resolution for clarity.
3- Line 93–94 – There are multiple poorly written sentences, where language need to be improved. These affect the overall clarity. I would suggest to the authors to have their paper proofread by an English native speaker. One example is “fluctuation of three-dimensional ordered subset expectation maximization (3D-OSEM) reconstruction of factors such as counts and reconstruction parameters.” What type of parameters are meant and unclear how counts impact reconstruction? Is image quality meant here or quantitation?

**Response:**

We thank you for the valuable comments on improving the clarity and precision of our manuscript.

Regarding the terminology for resolution (Comment 2) and the clarity of the subsequent sentences (Comment 3), we have rewritten the entire passage. The original text in the **Introduction** (previously Lines 91-94) has been revised to enhance accuracy and readability. We believe this revised text directly addresses your concerns by using the correct terminology ("poorer spatial resolution") and by clearly explaining how factors like counts and reconstruction parameters can influence the final image.

Revised manuscript: Pages 5-6, Lines 111-119:

*The quantitative accuracy of SPECT imaging have* *poorer spatial resolution compared to* *positron emission tomography images and the sensitivity of three-dimensional ordered subset expectation maximization (3D-OSEM) reconstruction algorithm to acquisition and reconstruction parameters. To establish optimal conditions for quantitative analysis, we used phantoms containing rod and spherical structures,* *as described in previous studies [14, 15].*

Furthermore, in line with your general comment, the entire manuscript has undergone professional proofreading by a native English-speaking scientific editor from Editage to enhance overall clarity, grammar, and style. We are confident that these comprehensive revisions have significantly improved the manuscript.

4- Line 105 –The photon energy for I-123 is 159 keV, not 158 keV.

**Response:**

Thank you for pointing out this unintended mistake. We have corrected the photon energy of I-123 from 158 keV to 159 keV throughout the manuscript. We apologize for this oversight. (Page 7, Line152)

5- Line 110 – The paper should specify the number of views around the 360-degree rotation, total scan time, and whether the acquisition was performed in continuous mode or step-and-shoot mode.

**Response:**

Thank you for this suggestion. We have added the requested details on the SPECT acquisition parameters to the manuscript. We have specified the number of views per 360-degree rotation, clarified how the total scan time was determined by the acquisition time per view, and stated that the acquisition was performed in step-and-shoot mode.

Revised manuscript: Page 7, Lines 154-159

*The matrix size was 128 × 128, with a zoom factor of 1.23. Voxel size was 3.90 × 3.90 × 3.90 mm. The sampling angle was 5° per step, resulting in 72 views over a full 360° rotation. A non-circular orbit was used with step-and-shoot acquisition mode. Acquisition time per detector was varied at 10, 30, 50, and 100 s/view, and the total scan time was adjusted accordingly.*

6- The paper does not describe how the system was calibrated to convert counts into activity concentration (Bq/ml). This is essential for understanding the reliability of the SUV values. It’s unclear (and unusual) why SUV values were evaluated with phantoms rather than focusing on quantitative accuracy. Normalizing against weight for SUV does not make sense for phantoms.

**Response:**

Thank you for your insightful comments on our methodology. We appreciate the opportunity to clarify our approach to system calibration and the rationale for using SUV in this phantom study. We have revised the manuscript to address each of your points.

First, regarding the system calibration method, you are correct that a detailed description is essential. We have now added a comprehensive explanation of our calibration procedure to the **Methods** section.

Revised manuscript: Page 6, Lines 136-146

*Prior to all phantom imaging, the established system calibration procedures were followed. The dose calibrator used to measure the radioactivity of ^123^I was cross-calibrated with a well counter. To verify the accuracy of the final in-phantom activity concentrations, we collected 1 mL aliquots from each prepared solution—for both the target inserts and background compartments of the JS-10 and NEMA body phantoms—and measured them using the cross-calibrated well counter immediately before and after phantom filling. Furthermore, a SPECT system calibration factor (e.g., Becquerel calibration factor, BCF) to convert image measured count data into activity concentration (Bq/mL) was established by imaging a cylindrical phantom (inner diameter: 16 cm, height: 15 cm, total volume: 3,016 mL) with a known radioactivity concentration of 123I, also determined by the calibrated dose calibrator.*

Second, concerning the rationale for evaluating SUV in a phantom study, our strategic goal is to provide a foundational bridge from the current clinical standard (the semi-quantitative H/M ratio) to the potential future use of fully quantitative SUV in ^123^I-MIBG SPECT. To clarify this objective and the significance of our findings, we have substantially expanded the "**Summary and implications**" and "**Conclusion**" sections.

Revised manuscript: Page 23, Lines 480-487

*The clinical implication of these findings is significant for the advancement of quantitative MIBG SPECT. By establishing technical parameters that ensure the stable and accurate quantification of not only high-uptake myocardial regions but also low-uptake background areas like the mediastinum, our work provides an essential foundation for enhancing the robustness of 3D-based quantitative metrics, such as the myocardial-to-mediastinal SUV ratio. This, in turn, has the potential to support more reliable and reproducible cardiac assessments in clinical practice.*

Revised manuscript: Page 23, Lines 498-501

*This work provides a foundational methodology for the robust quantification of ^123^I-MIBG SPECT. The optimized parameters established herein are expected to support future clinical research aimed at improving diagnostic accuracy and evaluating treatment effects in a wide range of cardiac and neurodegenerative disorders.*

Finally, addressing your point on the SUV calculation method in phantoms, we have clarified in the **Methods** section that our approach is consistent with standard practice.

Revised manuscript: Page 10, Lines 230-231

*Consistent with previous studies [19,20], the SUV was calculated by assuming a water density of 1 g/mL, thereby converting volume to an equivalent weight for normalization.*

7- The paper does not include repeated measurements to establish statistical significance of the results shown. The findings of this study thus remains of unclear significance.

**Response:**

Thank you for this critical comment regarding repeated measurements and statistical significance. We agree that repeat acquisitions are the gold standard for assessing inter-measurement variability. While our study protocol involved a single acquisition for each parameter set, we ensured the significance and reliability of our findings through a multi-faceted approach.

First, for our primary quantitative finding—dose linearity (DL)—we performed a direct statistical validation. We have now added a "**Statistical Analysis**" section to the **Methods** and explicitly state in the **Results** that all calculated Pearson's correlation coefficients were highly significant (p < 0.01). This provides strong statistical evidence for the stability of our quantitative method across all tested conditions.

Second, for all other metrics, we enhanced the robustness of each measurement using well-established techniques, consistent with prior foundational studies [14, 19]. Instead of relying on single-point values, metrics were derived from the average of multiple slices or 3D regions of interest. This established method minimizes the impact of random noise and operator bias. (Lines 191-196, Lines 224-229)

Finally, we have clearly framed the scope of our study and its single-acquisition design as a limitation. The primary objective of this study was to systematically map performance trends across a wide range of parameters, which is a necessary first step before conducting validation studies with repeated measurements. To be fully transparent with the reader, we have explicitly stated this in the revised **Limitations** section.

We are confident that our combination of direct statistical validation for our key results and robust measurement techniques based on established protocols provides a reliable and significant contribution.

Revised manuscript: Page 21, Lines 441-443

*This study has some limitations. First, it was conducted at a single facility using a single SPECT/CT device, and each imaging parameter set was evaluated with a single acquisition.*

Page 22, Lines 462-464

*However, to enhance robustness and minimize inter-operator variability, we evaluated multiple cross-sectional slices and performed three-dimensional multi-point analyses using two distinct phantom types [14,19]. In addition, …*

8- The paper should explain how the phantom was filled with activity and if the concentrations were based on realistic levels. Were these concentrations clinically realistic? Were the total counts consistent with clinical practice?

**Response:**

Thank you for your questions about the phantom filling procedure and the clinical realism of our activity concentrations and total counts.

First, we have added details on the phantom filling method to the **Materials and Methods** section as follows: Page 6, Lines 127-135

*For phantom preparation, we first measured a stock solution of ^123^I using a calibrated dose calibrator. To prepare the rod sections of the JS-10 phantom and the spheres of the NEMA IEC body phantom, we diluted the stock solution to achieve predetermined target activity concentrations, accounting for radioactive decay up to the time planned imaging start time. For the background compartments of both phantoms, we added a calculated volume of the stock solution—based on dose calibrator measurements—directly to the compartments. Thereafter, the phantoms were filled to their known volumes with water and mixed thoroughly to ensure uniform activity distribution.*

Regarding the clinical realism of the activity concentrations used, we have expanded the **Limitations** section to provide a more detailed explanation.

Pages21, Lines 445-452

*Second, the optimization was based on a phantom simulating a standard-sized individual (60 kg) who received a full dose of 111 MBq of ^123^I-MIBG. The activity concentrations used reflect clinically plausible scenarios: the background (mediastinal) concentration (1.42–1.73 kBq/mL) accounted for approximately 20–30% early excretion of the administered MIBG and assumed uniform distribution of the remaining activity [18,20].* *The hot sphere concentration (15.81 kBq/mL) was based on an estimated 1–2% of the effective in-body MIBG activity accumulating in the myocardium [27,28].*

To address the consistency of total counts with clinical practice, we have included projection count data as **Supplementary Material.** A reference to this supplementary information has been added to the end of the Results section. This will allow readers to compare these count levels with those typically encountered in their clinical practice.

The end of **Results,** Line 318

*The raw data used for the analyses are available as S1 Data*.

9- SUVmean in Figure 6 has very high error bars, which is unusual since SUVmean is typically stable over noise (average over a large area). The reason for this variability should be clarified. Also, the other schemes shown in Figure 6 do not have any error bars, making it difficult to interpret or compare the results. Including consistent error bars across all schemes and for all the metrics are essential.

**Response:**

Thank you for your insightful feedback on Figure 6. We agree that the original error bars were not appropriate for their context and could be misleading. We apologize for the confusion this caused.

The error bars originally represented the intra-VOI pixel standard deviation from a single measurement, not the inter-measurement variability. As you correctly point out, a consistent assessment of measurement variability is crucial. However, our study protocol involves a single acquisition for each condition, which makes it impossible to calculate the inter-measurement standard deviation or standard error that would be required for such an analysis.

To ensure clarity and scientific accuracy, we have removed the original error bars from Figure 6.

To address your valid concern about assessing the impact of noise, we have revised the manuscript to strengthen the narrative link between our quantitative noise analysis in Figure 5 and the SUV recovery trends shown in Figure 6. While our original **Discussion** section already touched upon the findings from both figures, the revised text now more explicitly guides the reader, clarifying how the noise characteristics detailed in Figure 5 (such as the CV) directly influence the SUV performance in Figure 6 (Lines 410-413).

We believe this approach clarifies the source of variability and provides a more robust interpretation of our results, fully addressing your concern.

Revised manuscript: Page 20, Lines 410-413

The stabilization of SUV values at 50 s/view aligns well with the substantial improvement in CNR observed up to this point in Figure 5. This suggests that a sufficient signal-to-noise ratio is a prerequisite for achieving robust SUV quantification.
